# How to Apply the Concept of Umwelt in the Evolutionary Study of Cognition

**DOI:** 10.3389/fpsyg.2018.02001

**Published:** 2018-10-17

**Authors:** Nereida Bueno-Guerra

**Affiliations:** Department of Psychology, Comillas Pontifical University, Madrid, Spain

**Keywords:** umwelt, cognition, evolution, methods, Uexküll, comparative psychology

Since our ancestors' time, human culture is plagued with anthropomorphizations, meaning the attribution of human features to non-human animals: deer embodying longevity in Japan or Western fables with talking animals are just some examples. This anthropomorphization was also enrooted in evolutionary psychology (see Wynne, 2007 for a historical review). The *Scala naturae*, the classical perspective of the tree of life, placed humans at the peak of a pyramid. This hierarchical polygon represented a progressive and cumulative race of cognitive capacities where humans crowned themselves as the superior species.

The hierarchical tradition dates back to the nineteenth century. The study of animals other than humans was so imbued with anthropomorphism that the nomenclature used for animals' sensorial physiology was based on how humans perceived the stimuli with their own sensorial system, despite essential differences between the animals and the humans in this respect (Wiese et al., 1990). This ended up largely biasing our understanding of other taxa and led some authors to stand up for a different approach to objective science, urging researchers to refer to species-specific characteristics as well as to each species' own evolutionary pathway without necessarily referring to humans as an ideal model (see the seminal paper by von Uexküll et al., 1899; and also Bethe, 1940). Within this period, Jakob Von Uexküll coined the concept of “Umwelt,” which paradoxically supported achieving objectivity through the study of subjectivity. In his own words: “All that a subject perceives becomes his perceptual world [Merkwelt] and all that he does, his effector world [Wirkwelt]. Perceptual and effector worlds together form a closed unit, the Umwelt” (von Uexküll, 1934/2010, p. 6).

*Merkwelt* meant a unique world composed of different perceptual worlds. Uexküll realized that life is shaped around senses, therefore species develop in a concrete framework of the reality. This idea, as Uexküll himself recognized (von Uexküll, 1920/2014), draws from the Kantian idea of the impossibility to grasp substantive knowledge beyond our senses, distinguishing between noumemon and phenomenon (Kant, 1781/1998). Hence, our in-set structure needs to be challenged when working with non-human animals. On the one hand, we should consider the sensory spectrum the animal can perceive to provide the most adequate elements and avoid/control those which we cannot perceive but the animal does (i.e., UV wavelength vision in birds). On the other hand, we should take the particularities of its perceptual organization into consideration to present relevant stimuli. Otherwise, we will know how the animal faces human situations, which are not the ecological situations that the species has overcome through evolution, and we will mistakenly conclude about the animal's cognition in the assumption that we share the same perceptual process. A pessimistic and relativistic approach would conclude that we could never get to know other Umwelten different from ours. However, we have the tech tools to investigate this information. For example, Kano and Tomonaga (2009) explored how humans and chimpanzees looked at pictures differently using eye-tracker technology, whereas Vallortigara et al. (1990) showed how the chick's “perceptual experience was different from that defined by the experimenter” (p. 98) varying the disposition of several elements around an object. Studies like this contribute to discover other animals' *Merkwelt*. Therefore, my suggestion when recruiting the materials for an animal cognition experimental set-up is first to select the sense through which the experimental subjects mainly rely on when receiving information to then providing to/avoiding the experimental stimuli in that sensory modality. This is crucial because we as humans could design more experiments based on sight and hearing than in chemical senses just because we rely more on them as well as failing to control some imperceptible stimuli to us. In Uexküll's words: “Incomprehensibly, we interpret the physical world as the only real just because it is built on the basis of our senses and actions. However, (…) the world for a Jacobean oyster, for example, is just movement. And the world for a bright jellyfish, is just electricity” (von Uexküll, 1920/2014, p. 92–93). Also, I recommend to carefully choose those elements that basic research showed to (not) have an effect on the animal's perceptual organization.

*Wirkwelt* meant that anatomy interplays with the species' ethogram, that is, with the array of actions that a species can perform. There is a popular vignette depicting some animals (a bird, a monkey, a penguin, an elephant, a fish, a seal and a dog) in front of a human interviewer in the middle of a savannah. The human says: “For a fair selection everybody has to take the same exam: please climb that tree.” The core of the joke lies in the fact that the monkey is the unique animal among that group that could climb the tree and pass the exam. This is a beautiful example of the devastating error we may commit in animal research if we do not consider what the tested species is able to do. Our “fair selection” should indeed be fair with the abilities of the animal we test. Usually, the best ally here is our imagination plus our ability to adapt the materials to the abilities of the experimental subject. Many researchers have conceived beautiful examples of *Wirkwelt*'s implementations in their experimental designs. For example, humans choose by pointing, but in absence of manipulative fingers, Mueller-Paul et al. (2014) implemented touch screens in which their red-footed tortoises “nose” to choose one of two figures. Similarly, the touchscreen-nose-method was also applied when testing whether dogs preferred human facial expressions of happiness (Müller et al., 2015). Perry and Barron (2013) studied awareness of certainty in honeybees (*Apis mellifera*). To allow subjects showing preference between two tasks, the authors simply placed them in different tunnel-like chambers, so that the bee-subjects could freely fly through one or another. In the cases above, the lack of pointing was replaced by approaching, touching or flying, which were part of those animal's ethograms. Therefore, in line with Cook (1993), my first suggestion when designing the tasks for an animal cognition experimental set-up is to observe the animal, elaborate and study its ethogram. After that, the researcher should carefully think of his available technology plus which potential actions should be more appropriate to measure according to the animals' ethogram.

So far, I have discussed Uexküll's concept of Umwelt, split into the perceptual (*Merkwelt*) and motor (*Wirkwelt*) spheres of each species. My opinion, however, is that Uexküll's Umwelt should be broadened to include the social sphere (*Sozialwelt*), meaning how individuals of this species usually interact with conspecifics. For example, taking the social sphere into account was found to be essential in primate research. Cooperative set-ups, where chimpanzees could solve a task by helping each other, failed to consistently yield positive results. This was shocking, perhaps because human societies are grounded on cooperative bonds which have been shaped through evolution (Tomasello and Vaish, 2013). However, that evolutionary pathway may not be identical in other species. Indeed, the change of paradigm from cooperative to competitive set-ups (Hare et al., 2001) allowed researchers to study a vast array of different capacities in chimpanzees, thus making primatological studies more ecologically valid (Bates and Byrne, 2007). I can also provide an example of how social dynamics may influence the interpretation of the results. Together with some colleagues (data submitted and available upon request) we wanted to explore morality in non-human primates. In our set-up, that implied presenting “good” and “bad” experimenters to chimpanzees and let them choose among them. Interestingly, we had no homogeneous general results, however young males consistently chose the bad experimenter. Revisiting the underlying social meaning of the actions we have presented, we realized that we had defined “bad experimenter” as someone entering in a room and hitting a third individual whereas “good experimenter” was someone interrupting the fight and consoling the victim. Mostly all humans would have agreed with these actions being bad and good, respectively. However, would not it be possible that young males could have perceived the bad experimenter as good because during adolescence juveniles show preference for potential allies in future fights (a strong individual that hits others)? If we had not realized about this, we could have generalized the main result to the chimpanzee-species, rather than differentiating between adult and adolescent chimpanzees. Moreover, the interplay of hormones in social interactions should be also considered in those species which rely on olfactory cues or show seasonal behavior changes, as it has shown its powerful effect on different social behaviors (i.e., cooperation: Madden and Clutton-Brock, 2011; infant care: Finkenwirth et al., 2016). Finally, individual differences may also be known in advance or reported to avoid delusive generalizations (Stevens, 2010). Tasks and interpretations, therefore, should consider the social, lifespan, hormonal, and temperamental characteristics of the species/individuals.

Given the text above, in my opinion, to design an ecological experimental set-up, researchers should take into account three specific-species spheres: (1) perceptual sphere, (2) ethogram sphere, and (3) social sphere. Since getting to know the species-specific main characteristics is a must of experimental researchers, further research should consider conducting prior descriptive investigations.

The previous suggestions might be considered obvious, however, they become crucial to reconcile different Umwelten in multi-species comparative experimental research methods (Bueno-Guerra and Amici, 2018). Can we compare results from species with different *Merkwelt*? It might depend on the equivalence of the impact that the stimulus has for the species involved plus our research question. For example, if we are interested in knowing the satiation limit for each species, the same food stimulus (in case it is similarly rewarding for both species) plays the same function for them (i.e., nutrition) and therefore it might not be crucial the fact that they perceive the food differently. However, if the stimulus is the cue used to highlight the presence of some learning condition (i.e., color brings reward), then it might be mandatory to show the stimulus at the least common sensory spectrum window among all the species involved to avoid unfair advantages. In case finding this common window was problematic, then using different sensory stimulus (i.e., low/high sound and red/green color), assuring that no species is being provided an extra cue from other sense, might make the results still comparable. It would be analogous as if Braille dots, perceived by touching, and words, perceived by seeing, were used for two humans at a comprehension task. The main caveat would arise if variations of the stimulus played a role in the way the subjects process the information, since researchers would need to show that the species share similar cognitive perceptual organization regardless of the sensory modality employed. Can we compare results from species with different *Wirkwelt*? Not being able to combine different ethograms into one common action to be measured should not prevent from comparison between species if the chosen action per species is functionally equivalent in all of them. For example, humans of different cultural backgrounds express rejection with different actions: nodding the head up and down (Bulgary) is equivalent to say “nein” (Germany) and to moving the index from left to right (Spain). Can we compare results from species with different *Sozialwelt*? In these cases, the comparison is usually the research question [i.e., the role of oxytocin in solitary and social species, (Anacker and Beery, 2013)].

Yet, solving these questions exceeds the aims of this manuscript. However, they may occupy a prominent place in the methodological discussion of how Umwelt should be tackled in comparative psychology research. Indeed, it is worth asking whether a mandatory paragraph/supplementary material about how the three Umwelt spheres were considered in any multispecies study should be requested by default from specialized journals when considering it for publication, just as the ethical statement is requested. This would help researchers (and reviewers) not to forget any species' particularity that may mediate their design as well as it would enhance the strength of their conclusions.

## Author contributions

The author confirms being the sole contributor of this work and approved it for publication.

### Conflict of interest statement

The author declares that the research was conducted in the absence of any commercial or financial relationships that could be construed as a potential conflict of interest.
